# Improvement of Output Characteristics of Dual-Loss-Modulated Laser with Heterostructure Saturable Absorber at 1.3 μm

**DOI:** 10.3390/ma19112283

**Published:** 2026-05-28

**Authors:** Gang Zhang, Lanying Yang, Wenning Wang, Jie Weng, Fen Bai, Zhiyong Jiao

**Affiliations:** College of Science, China University of Petroleum (East China), Qingdao 266580, China; s23090003@s.upc.edu.cn (L.Y.); jiaozhy@upc.edu.cn (Z.J.)

**Keywords:** Q-switched laser, dual-loss-modulated, 1.3 μm, heterostructure saturable absorber

## Abstract

Dual-loss-modulated Q-switched lasers at 1.3 μm are achieved by using both electro-optic modulators (EOMs) and different saturable absorbers (SAs). Laser pulses with a width of 7.75 ns at 60 kHz and a peak power of 4.07 kW at 20 kHz can be obtained in lasers with both an EOM and a MoS_2_/WS_2_ heterostructure SA. Compared with those generated in lasers with a MoS_2_ SA under the same repetition rates, the pulse width is compressed by 37.7% (at 60 kHz), and the peak power is increased by 80.9% (at 20 kHz). The results show that dual-loss-modulated technology makes it easier to achieve pulses with a duration of several nanoseconds. Meanwhile, compared to single-material SAs, the heterostructure SA helps to generate pulses with a short duration and high peak power in 1.3 μm lasers.

## 1. Introduction

High-peak-power and narrow-width pulses at 1.3 μm can be applied in laser medicine, information storage, micro-machining, coherent communication, and other fields [1,2,3,4,5]. Both active and passive modulation can be utilized to achieve Q-switched (QS) pulses. Compared with single active/passive modulated technology, dual-loss-modulated technology has attracted extensive attention due to its advantages of compressing the pulse width, increasing the pulse, and enhancing stability.

Transition metal chalcogenides (TMCs) have aroused great interest among researchers due to their excellent physical and chemical properties [6,7,8]. The use of TMCs in 1.3 μm lasers has also been investigated. In 2016, K. Wang reported a 1.3 μm passive QS laser using a MoS_2_ SA [9]. In 2019, our group realized QS pulses by using a MoS_2_ SA [10] and a WS_2_ SA [11]. It can be seen that QS lasers at 1.3 μm based on TMCs can generate pulses with a pulse duration exceeding 100 ns. Then, heterostructure SAs prepared by using different 2D materials were studied in order to obtain better output characteristics [12,13,14]. In 2015, Zhao et al. used a MoS_2_/graphene heterostructure SA to realize 1.06 μm QS pulses [15]. Sun et al. realized mode-locked pulses at 1037 nm by using a graphene/MoS_2_ heterostructure SA [16]. In 2022, Liu et al. realized stable output of a 1.06 μm laser using a MoS_2_/MoSe_2_ heterostructure SA [17]. However, QS lasers at 1 μm with a heterostructure SA can only generate pulses with a duration of tens of nanoseconds. Determining how to compress the pulse width and obtain optimized laser characteristics is still an important research point.

Dual-loss modulation technology combines active and passive modulation to enhance the pulse stability and compress the pulse width. All-solid-state lasers with active Q-switch and 2D-material SAs have been reported. As early as 2016, Tang et al. reported a QS and mode-locked laser with an EOM and graphene. Single mode-locked pulses at 532 nm were realized [18]. In 2017, a doubly QS Nd:GGG laser with both an active modulator and a MoS_2_ SA was realized [19]. In 2021, Niu et al. realized a dual-loss-modulated Tm:YAP laser with both an EOM and an Sb_2_Te_3_ SA, highlighting the advantages of dual-loss modulation technology [20]. In 2022, our research group achieved a QS and mode-locked laser with a narrower envelope width using an acousto-optic Q-switch and a MoSe_2_ SA [21]. However, to the best of our knowledge, research on dual-loss-modulated QS lasers at 1.3 μm with both an EOM and a heterostructure SA has not been reported.

In this paper, we demonstrate a stable 1.3 μm QS laser using both an EOM and a MoS_2_/WS_2_ heterostructure SA. This is the first time an EOM has been combined with a MoS_2_/WS_2_ heterostructure SA to realize a dual-loss-modulated QS laser at 1.3 μm. The EOM enhances the pulse stability, while both the EOM and the heterostructure SA compress the pulse width. Compared with those obtained in an electro-optic QS laser and a dual-loss-modulated QS laser with a MoS_2_ SA, the pulse width of the dual-loss-modulated QS laser with a MoS_2_/WS_2_ SA is compressed by 62.3% and 37.7%, respectively, revealing the superior pulse compression capability of the MoS_2_/WS_2_ heterostructure SA.

## 2. Characterization of SAs

The MoS_2_/WS_2_ heterostructure SA used in this experiment was prepared by a wet transfer method described in ref. [22]. The MoS_2_ and WS_2_ films were both prepared by chemical vapor deposition (CVD) [23].

**Figure 1:**
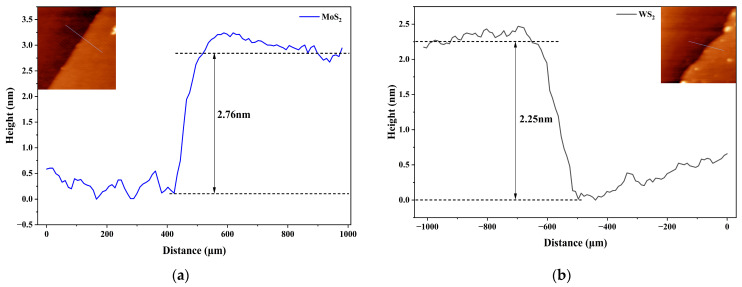
Characterization of the MoS_2_ and WS_2_ films: (**a**) height distribution and AFM image of the MoS_2_ film (the inset); (**b**) height distribution and AFM image of the WS_2_ film (the inset).

Atomic Force Microscopy (AFM) was employed to measure the thickness and morphology of the MoS_2_ and WS_2_ films. The AFM image and corresponding height distribution of the MoS_2_ film are shown in Figure 1a. The average thickness measured from the height profile was ~2.76 nm, indicating that the MoS_2_ SA used in the experiment was approximately 4 layers thick. The AFM image and corresponding height distribution of the WS_2_ film are shown in Figure 1b. The average thickness measured from the height profile was ~2.25 nm, indicating that the WS_2_ SA used in the experiment was approximately 3 layers thick.

**Figure 2:**
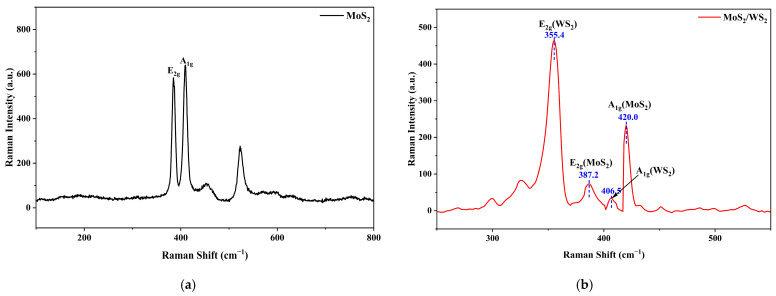
Raman spectra of SAs: (**a**) few-layer MoS_2_ SA; (**b**) MoS_2_/WS_2_ heterostructure SA.

The Raman spectra of the few-layer MoS_2_ SA and the MoS_2_/WS_2_ heterostructure SA used in this experiment are shown in Figure 2. As shown in Figure 2a, the two characteristic peaks E_2g_ and A_1g_ of the few-layer MoS_2_ SA were located at 384.8 cm^−1^ and 409.5 cm^−1^, respectively. In the MoS_2_/WS_2_ heterostructure (Figure 2b), the characteristic peak A_1g_ of MoS_2_ SA was located at 420.0 cm^−1^, and the characteristic peak E_2g_ was located at 387.2 cm^−1^. The two peaks centered at 355.4 cm^−1^ and 406.5 cm^−1^ are the characteristic peaks of the WS_2_ SA, which are attributed to the coupling within the heterostructure. According to the results reported in references [24,25], the few-layer MoS_2_ SA used here was 4 layers thick, and the WS_2_ SA was 3 layers thick. When the number of layers is small, the modulation depth is low and the pulse compression capability is weak. Although an excessively large number of layers provides a high modulation depth, the non-saturable loss increases significantly, leading to a decrease in output power and a broadening of the pulse width [24].

**Figure 3:**
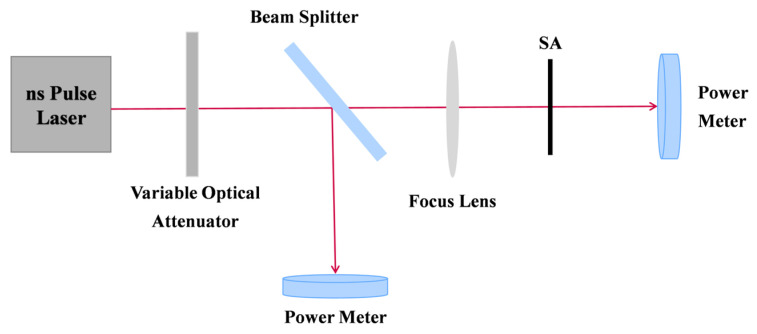
Experimental setup for a balanced dual-detector measurement system.

**Figure 4:**
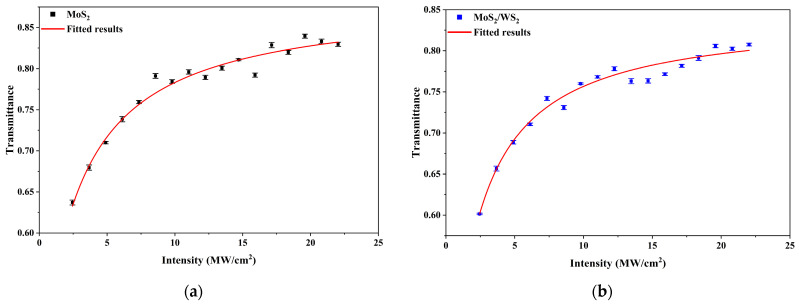
Nonlinear transmittance curves: (**a**) MoS_2_ SA; (**b**) MoS_2_/WS_2_ heterostructure SA.

To further determine the saturable absorption properties of the MoS_2_ SA and MoS_2_/WS_2_ heterostructure SA at 1342 nm, a balanced dual-detector measurement system, as shown in Figure 3, was used for detection. The laser source was a homemade electro-optic QS laser with a wavelength of 1342 nm, a pulse duration of 10 ns, and a repetition rate of 60 kHz. The variation of transmittance with incident light intensity is fitted by

(1) $$T(I)=1-\frac{\Delta T}{1+I/I_{sat}}-T_{ns}$$

where Δ*T* is the modulation depth, *I_sat_* is the saturation intensity, and *T_ns_* is the non-saturable loss [26]. The nonlinear transmission curves of the two samples are shown in Figure 4. The saturation intensity and modulation depth of the MoS_2_ SA were (5.76 ± 0.7) MW/cm^2^ and (19.2 ± 0.3)%, respectively, while those of the MoS_2_/WS_2_ heterostructure SA were (5.42 ± 0.5) MW/cm^2^ and (20.5 ± 0.2)%, respectively.

The vertical heterostructure formed between MoS_2_ and WS_2_ facilitates interlayer charge transfer and enhances light–matter interaction, thereby increasing the modulation depth. Secondly, the type II band alignment in the heterostructure helps suppress the recombination of photogenerated carriers and accelerates the recovery time of the saturable absorber, which is beneficial for generating shorter pulses. Additionally, the higher carrier mobility of WS_2_ compensates for certain defects in MoS_2_, reducing the saturation intensity [27]. The modulation depth of the MoS_2_/WS_2_ SA is only about 1.3% higher than that of the MoS_2_ SA, and the saturation intensity is about 5.9% lower. This difference is amplified during the Q-switching process in the actual cavity. A lower saturation intensity means that the SA is more easily bleached, resulting in lower intracavity loss under the same pumping conditions. A slightly higher modulation depth further enhances the amplitude of loss modulation, leading to faster pulse buildup and narrower pulse width [28]. This indicates that the laser with a MoS_2_/WS_2_ heterostructure SA has the potential to generate narrower pulses.

## 3. Experimental Setup

**Figure 5:**
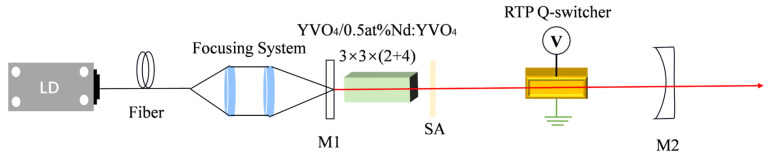
Schematic of dual-loss-modulated QS YVO_4_/Nd:YVO_4_ laser.

Figure 5 presents a schematic of the QS laser applied in this experiment. A semiconductor laser with a central wavelength of 808 nm was used as the pump source. The gain medium was an YVO_4_/Nd:YVO_4_ bonded crystal with a cross-sectional size of 3 mm × 3 mm and a length of (2 + 4) mm, with Nd^3+^ doping concentrations of 0 at% and 0.5 at%, respectively. The two end faces were both coated with high-transmission (HT) film at 808 nm and 1342 nm. To reduce the thermal effect of the YVO_4_/Nd:YVO_4_ crystal, a circulating water-cooling system was used to cool the laser crystal, with a water temperature of 20.6 °C. A RbTiOPO_4_ (RTP) electro-optic Q-switch (Fastpulse, Saddle Brook, NJ, USA) served as the active modulator. The MoS_2_ SA and the MoS_2_/WS_2_ heterostructure SA served as the passive modulator.

A simple plane–concave cavity with a cavity length of 115 mm was adopted. The input mirror M1 was a plane mirror with HT coatings at 808 nm and 1064 nm and a high-reflection (HR) coating at 1342 nm. The output mirror M2 was a concave mirror with a curvature radius of 200 mm and a transmittance of 12% at 1342 nm. The MoS_2_ SA or the MoS_2_/WS_2_ heterostructure SA was vertically (perpendicular to the optical axis) inserted between the gain medium and the RTP Q-switch, close to the gain medium. The optical spectrum characteristics were analyzed by an optical spectrometer (AQ6370D, Yokogawa, Tokyo, Japan). The average powers were given by a laser power meter (LaserPoint, Milan, Italy). The pulses were recorded by an InGaAs fixed-gain amplified detector (PDA015C, Thorlabs, Newton, NJ, USA) with a rise time of 1.0 ns and an oscilloscope (MSO64B, Tektronix, Beaverton, OR, USA) with a bandwidth of 4 GHz, corresponding to a rise time of approximately 100 ps.

## 4. Experimental Results and Discussion

When the electro-optic Q-switch was off and the SA was not inserted into the resonator, the laser operated in continuous wave (CW) mode with a threshold power of 2.4 W. After the EOM was turned on, an electro-optic QS laser was realized, and the laser output threshold increased to 2.7 W. To optimize the laser output characteristics, the MoS_2_ SA and the MoS_2_/WS_2_ heterostructure SA were introduced into the electro-optic QS laser, forming a dual-loss-modulated QS laser. The introduction of the MoS_2_ SA or the MoS_2_/WS_2_ heterostructure SA increased the loss, thereby raising the laser threshold. Stable pulses were obtained when the threshold power reached 3 W.

To evaluate the long-term stability of the laser, the average output powers were continuously recorded for 2 h at a repetition rate of 60 kHz and a pump power of 11 W, with sampling every 10 min. The results showed that the output power fluctuation was less than ±2.5%. Moreover, after the operation, the nonlinear transmission curve of the SA was remeasured, and no significant changes in modulation depth or saturation intensity were observed, indicating that the MoS_2_ SA and MoS_2_/WS_2_ heterostructure SA had good damage resistance under continuous high-power irradiation.

**Figure 6:**
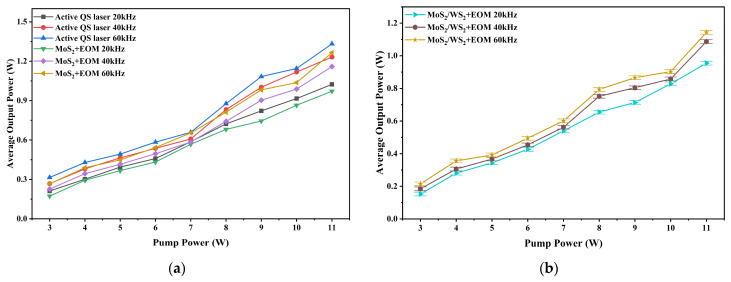
Relationship between average output power and pump power: (**a**) active QS laser and dual-loss-modulated QS laser with MoS_2_ SA; (**b**) dual-loss-modulated QS laser with MoS_2_/WS_2_ SA (with error bars).

Based on the results of this experiment and previous reports, it can be seen that the stability of QS pulses using only SAs is lower than that of electro-optic QS pulses. Therefore, this experiment focused on the output characteristics of electro-optic QS lasers and dual-loss-modulated QS lasers. In order to understand the influence of the electro-optic modulation frequency on laser output characteristics, QS lasers with different electro-optic modulation rates (20 kHz, 40 kHz, and 60 kHz) were realized. Figure 6 shows that the average output power increased with the pump power. For the dual-loss-modulated laser constructed with the MoS_2_/WS_2_ heterojunction SA, systematic error analysis and uncertainty evaluation of the average output power were carried out, and it was determined that the measurement uncertainty of the average output power of the laser did not exceed ±0.011 W. Under otherwise identical conditions, the laser achieved the highest average output power at 60 kHz. Within the tested range, a higher repetition rate led to a higher average output power.

**Figure 7:**
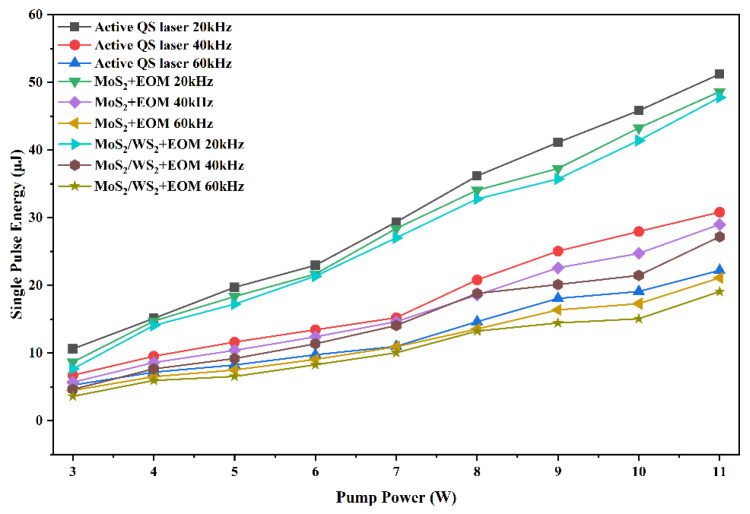
Relationship between single-pulse energy and pump power.

Figure 7 shows the single-pulse energies calculated from the repetition rate and the average output power. It can be seen that a lower repetition rate helped to increase the single-pulse energy, and the pulse energy increased with pump power in the same laser. At a repetition rate of 20 kHz and a pump power of 11 W, 51.2 μJ was obtained in the active QS laser, while about 47.8 μJ was obtained in the dual-loss-modulated QS laser with the MoS_2_/WS_2_ SA. Compared with the single-pulse energies obtained at 3 W, the highest single-pulse energies at 20 kHz for the active QS laser, the dual-loss-modulated QS laser with the MoS_2_ SA, and the dual-loss-modulated QS laser with the MoS_2_/WS_2_ heterostructure SA increased by 4.23, 4.74, and 5.23 times, respectively, when the pump power was raised to 11 W.

**Figure 8:**
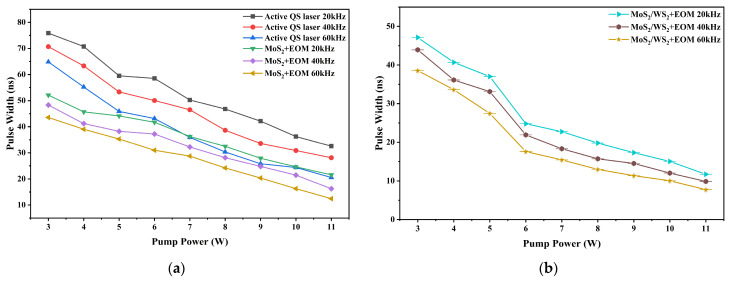
Relationship between pulse width and pump power: (**a**) active QS laser and dual-loss-modulated QS laser with MoS_2_ SA; (**b**) dual-loss-modulated QS laser with MoS_2_/WS_2_ SA (with error bars).

**Figure 9:**
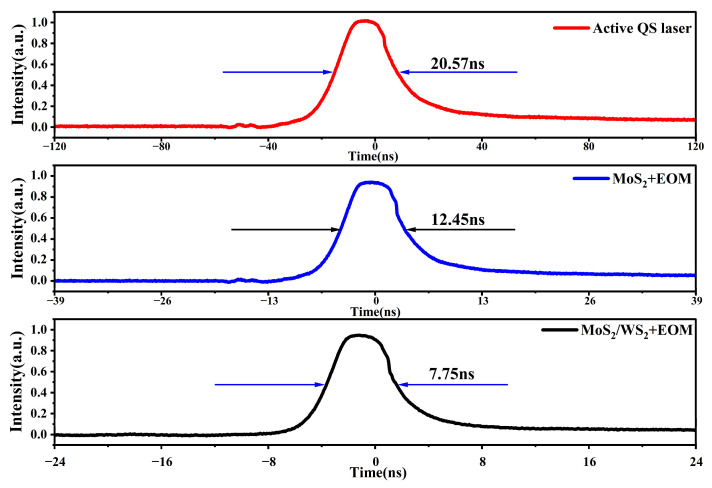
Pulse comparison of active QS laser and dual-loss-modulated QS lasers.

The relationship between the single-pulse width and pump power is shown in Figure 8. The pulse width of the dual-loss-modulated QS laser with the MoS_2_/WS_2_ SA was significantly compressed compared with those of the active QS laser and the dual-loss-modulated QS laser with the MoS_2_ SA. For the dual-loss-modulated laser with the MoS_2_/WS_2_ SA, systematic error analysis and uncertainty evaluation of the pulse width were carried out, and it was determined that the measurement uncertainty of the pulse width of the laser did not exceed 0.001 ns. At 11 W and 60 kHz, the narrowest pulse width of approximately 7.75 ns was obtained in the dual-loss-modulated QS laser with the heterostructure SA, compressed by 62.3% compared with that of the active QS laser and by 37.7% compared with that of the dual-loss-modulated QS laser with the MoS_2_ SA. The results show that dual-loss modulation using both an EOM and a heterostructure SA can significantly compress the pulse width. Figure 9 records the pulse width comparison of the active QS laser and the MoS_2_ SA and MoS_2_/WS_2_ heterostructure SA dual-loss QS lasers at 60 kHz, where the dual-loss modulation exhibits a more stable pulse train. The experimental pulse performance indicates that the interaction between the EOM and the SA is essentially an active–passive synergistic modulation mechanism. This synergistic modulation method compresses the pulse width, improves the symmetry of the pulse waveform, and enhances the stability of the pulse train.

**Figure 10:**
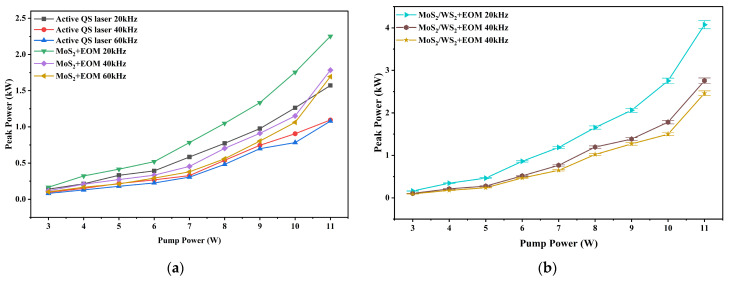
Relationship between peak power and pump power for different lasers: (**a**) active QS laser and dual-loss-modulated QS laser with MoS_2_ SA; (**b**) dual-loss-modulated QS laser with MoS_2_/WS_2_ SA (with error bars).

**Figure 11:**
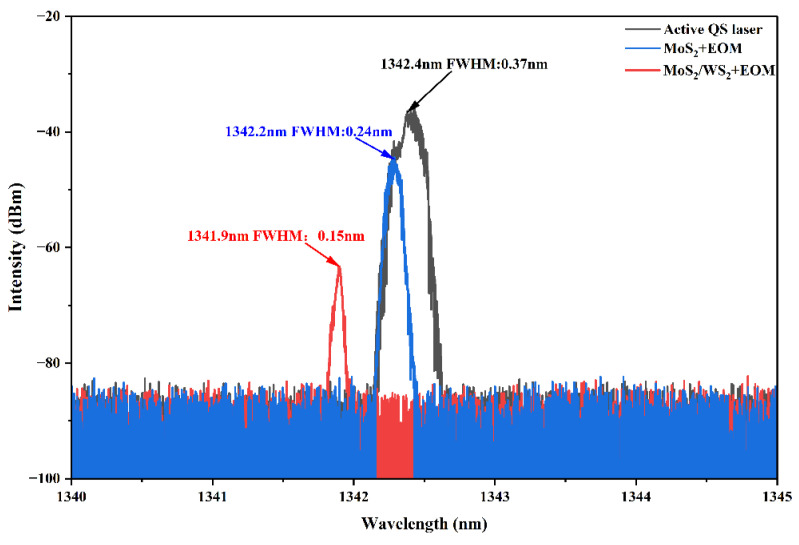
Output spectra for different QS lasers.

The peak powers were calculated by using the single-pulse energies and pulse durations. The relationship between the peak powers and pump powers is given in Figure 10. At 20 kHz and 11 W, the peak power of the QS laser with both an EOM and the heterostructure SA was 4.07 kW, increasing by 80.9% compared with that of the QS laser with an EOM and the MoS_2_ SA and by a factor of 1.59 compared with that of the active QS laser. At 60 kHz, the peak power of the QS laser with an EOM and the heterostructure SA increased by 45.2% compared with that of the QS laser with an EOM and the MoS_2_ SA. The above results indicate that the use of the MoS_2_/WS_2_ heterostructure SA can significantly improve the pulse peak power at different repetition rates. The spectra of the active QS laser and dual-loss-modulated QS lasers are shown in Figure 11. The peak wavelengths were 1342.4 nm, 1342.2 nm, and 1341.9 nm. According to references [29,30], the spectral pulse shift is a result of the interaction between the physical properties of the SA and the parameters of the laser resonator. Meanwhile, it can be seen that the full width at half-maximum (FWHM) was 0.15 nm for the dual-loss-modulated QS laser with the heterostructure SA, leading to better monochromaticity compared with the active QS laser and the dual-loss-modulated QS laser with MoS_2_.

Table 1 summarizes the performance of 1.3 μm lasers under different modulation methods based on TMDs. The data show that the dual-loss-modulated technique, which combines an EOM with a MoS_2_/WS_2_ heterostructure SA, has a significant modulation advantage, enabling narrower pulse widths and higher peak powers.

## 5. Theoretical Analysis

For a Nd:YVO_4_ laser using Nd^3+^ as the activation ion, the energy levels involved in the laser transition belong to a four-level system [32]. In this study, using YVO_4_/Nd:YVO_4_ as the gain medium, the dual-loss-modulated laser based on the MoS_2_/WS_2_ heterostructure SA adopts a four-level system, and its dual-loss-modulated rate equations are as follows [33]:

(2) $$\frac{\mathrm{dN}}{\mathrm{dt}}=R_{p}-\frac{N}{\tau }-\gamma_{g}N\phi$$

(3) $$\frac{dn_{s}}{\mathrm{dt}}=\frac{\sigma_{\mathrm{gs}}c}{V}\left(n_{s0}-n_{s}\right)\phi -\frac{n_{s}}{\tau_{s}}$$

(4) $$\frac{d\phi }{\mathrm{dt}}=\left(\gamma_{g}N-\gamma_{s}\left[\sigma_{\mathrm{gs}}\left(n_{s0}-n_{s}\right)+\sigma_{\mathrm{es}}n_{s}\right]-\frac{1}{\tau_{c}\left(t\right)}\right)\phi +\beta \frac{N}{\tau }$$

where *N* is the population inversion density, $R_{p}=\eta_{p}P_{p}/(h\nu_{p}V)$, *R_P_* is the pump rate, *η_p_* is the laser pumping efficiency, and *V* is the mode volume in the gain medium. $\gamma_{g}=\sigma_{g}c/V$ is the stimulated emission coefficient of the gain medium, *σ_g_* is the stimulated emission cross-section of the gain medium, and *c* is the speed of light in vacuum. *n_s_* is the excited-state population density of the absorber, *n_s_*_0_ is the initial population density of the absorber, and *τ_s_* is the excited-state lifetime of the absorber. *ϕ* is the photon density, *σ_gs_* is the ground-state absorption cross-section of the absorber, *σ_es_* is the excited-state absorption cross-section of the absorber, *τ* is the upper-state lifetime, and *β* is the spontaneous emission factor. *τ_c_*(*t*) is the cavity photon lifetime (controlled by the electro-optic switch) [34]:

(5) $$\tau_{c}(t)=\{\begin{array}{c} \;\tau_{c,\mathrm{off}}\;(\min)\;\;\;t<t_{\mathrm{EO}}\\ \tau_{c,\mathrm{on}}=L/c\delta_{T}\;\;\;\;\;t\geq t_{\mathrm{EO}} \end{array}$$

If pumping and spontaneous emission during the pulse are neglected, integration yields the single-pulse energy formula:

(6) $$E=h\nu \frac{\delta_{\mathrm{oc}}}{\delta_{T}}VN_{\mathrm{th}}\ln\left(\frac{N_{i}}{N_{f}}\right)$$

$N_{\mathrm{th}}=\delta_{T}/\left(\sigma_{g}l_{g}\right)$ is the threshold inversion density for fixed loss, where *δ_T_* is the total single-pass loss, and *l_g_* is the length of the gain medium. *hυ* is the photon energy, and *δ_oc_* is the output coupling loss. *N_i_* and *N_f_* represent the inversion population densities at the start and end of the pulse, respectively, satisfying the energy storage equation and the energy extraction equation:

(7) $$N_{i}=R_{p}\tau \left(1-e^{-T/\tau }\right)+N_{f}e^{-T/\tau }$$

(8) $$N_{i}-N_{f}-n_{s0}\left[1-{\left(\frac{N_{f}}{N_{i}}\right)}^{z}\right]-N_{\mathrm{th}}\ln\left(\frac{N_{i}}{N_{f}}\right)=0$$

where *z* is the cross-section ratio. The peak power formula is

(9) $$P_{m}=h\nu \frac{\delta_{\mathrm{oc}}}{\delta_{T}}\frac{V}{\tau_{c}}\left[N_{i}-N_{p}-n_{s0}\left(1-{\left(\frac{N_{p}}{N_{i}}\right)}^{z}\right)-N_{\mathrm{th}}\ln\left(\frac{N_{i}}{N_{p}}\right)\right]$$

where *N_p_* is the peak inversion density, satisfying $N_{p}-zn_{s0}{\left(\frac{N_{p}}{N_{i}}\right)}^{z}-N_{\mathrm{th}}=0$.

The optimal single-pulse energy and peak power at different repetition rates can be calculated from the parameters given in Table 2 at a pump power of 11 W. The theoretical calculation results are shown in Table 3. The calculated values are in good agreement with the experimental results, indicating that dual-loss modulation with both the MoS_2_/WS_2_ heterostructure SA and the EOM is reasonable and capable of achieving pulse width compression.

## 6. Conclusions

A dual-loss-modulated QS Nd:YVO_4_ laser with an EOM and different TMD SAs was demonstrated. The EOM was used to improve the stability of QS pulses, and dual-loss modulation was used to compress their duration. Under the same pumping conditions, narrower pulse widths and higher peak power were realized in the dual-loss-modulated QS laser than in the active QS laser. At a repetition rate of 60 kHz and a pump power of 11 W, the pulse width of the dual-loss-modulated QS laser with the heterostructure SA was compressed to 7.75 ns, with a peak power of 2.46 kW. Compared with the dual-loss-modulated QS laser with the MoS_2_ SA, the pulse width was reduced by 37.7%, and the peak power increased by 45.2%. The experimental results not only indicate that the combination of an EOM and an SA can achieve stable laser output but also demonstrate that compared with a single-material SA, the heterostructure SA makes it easier to optimize laser output characteristics, leading to a narrow pulse width and high peak power.

## Figures and Tables

**Table 1:** Output characteristics for lasers with TMDs at 1.3 μm.

Modulated Device	Pulse Width	Peak Power	Average Output Power	Ref
MoS_2_ SA	188 ns	13.45 W	144 mW	[9]
MoS_2_ SA	140 ns	23.8 W	1.1 W	[10]
WS_2_ SA	550 ns	10.1 W	538.3 mW	[11]
ReS_2_ SA	403 ns	0.9 W	78 mW	[31]
AOM + MoSe_2_	155 ns	48 kW	0.55 W	[21]
EOM + MoS_2_	12.45 ns	2.46 kW	1.27 W	This work
EOM + MoS_2_/WS_2_	7.75 ns	1.69 kW	1.14 W	This work

**Table 2:** Parameters in theoretical calculations.

Parameters	Values	Parameters	Values
*hν*	1.481 × 10^−19^ J	*hν_p_*	2.46 × 10^−19^ J
*η_p_*	0.52	*τ*	100 μs
*L*	0.115 m	*l_g_*	0.006 m
*T_oc_*	12%	*δ_oc_* = −ln(1 − *T_oc_*)	0.128
*f*	20, 40, 60 kHz	*δ_in_*	0.026
*A*	1.39 × 10^−7^ m^2^	*V = A* × *l_g_*	8.3 × 10^−10^ m^3^
*β*	~10^−5^	*σ_g_*	2.75 × 10^−22^ m^2^
*σ_gs_*	1.2 × 10^−21^ m^2^	*σ_es_*	2.4 × 10^−22^ m^2^
*n_s_*_0_	4.5 × 10^28^ m^−3^		

**Table 3:** Theoretical values and experimental results for output characteristics at different repetition rates with a pump power of 11 W.

Repetition Rates		20 kHz	40 kHz	60 kHz
Single-pulse energy	Theoretical values/μJ	48.32	27.64	19.51
	Experimental values/μJ	47.75	27.18	19.07
Peak power	Theoretical values/kW	4.21	2.95	2.67
	Experimental values/kW	4.07	2.76	2.46

## Data Availability

The original contributions presented in this study are included in the article. Further inquiries can be directed to the corresponding author.
